# Propolis improves pregnancy outcomes and placental oxidative stress status in streptozotocin-induced diabetic rats

**DOI:** 10.1186/s12906-018-2391-6

**Published:** 2018-12-05

**Authors:** Umar Zayyanu Usman, Ainul Bahiyah Abu Bakar, Mahaneem Mohamed

**Affiliations:** 1 Department of Physiology, College of Health Sciences, Usman Danfodiyo University, Sokoto, Nigeria; 2 Department of Physiology School of Medical Sciences, Universiti Sains Malaysia, 16150 Kubang Kerian, Kelantan Malaysia; 3 Unit of Integrative Medicine, School of Medical Sciences, Universiti Sains Malaysia, 16150 Kubang Kerian, Kelantan Malaysia

**Keywords:** Diabetes, Propolis, Pegnancy outcomes, Oxidative stress

## Abstract

**Background:**

This study assessed the effects of propolis alone or combined with insulin on maternal status, pregnancy outcomes and placental oxidative stress in streptozotocin-induced diabetic rats.

**Methods:**

Forty female rats were randomly assigned into five groups (*n* = 8/group) i.e. non-DM (non-diabetes), DM (diabetes), DM + Propolis (diabetes on propolis orally); DM + Insulin (diabetes on insulin subcutaneously) and DM + Combined (diabetes on propolis and insulin) groups. Propolis and insulin were given at 300 mg/kg/day orally and 5.0 IU/kg/day subcutaneously, respectively, for 4 weeks.

**Results:**

Fasting blood glucose, conception period, implantation losses, foetal blood glucose and placental oxidative stress markers such as malonaldehyde and protein carbonyl were significantly higher while maternal weight gain, foetal body weight and total antioxidant capacity were significantly lower in DM group compared with non-DM group. These changes were significantly improved in rats treated with propolis or insulin alone with greater significant effects in rats treated with both propolis and insulin.

**Conclusion:**

This study may suggest the protective effects of propolis against DM-induced impaired pregnancy outcomes and placental oxidative stress with greater effects when combined with insulin.

## Background

There is an increase in the prevalence of diabetes mellitus (DM) which is projected to increase up to 642 million by 2040. Its effect on the patient socio-economy, physical and medical state has become a major concern globally [1]. Diabetes-induced hyperglycaemic changes have been found to be potentially detrimental to intrauterine foetal development [2]. In an experimental rat, maternal hyperglycaemia-exposed foetus has septal hypertrophy [3]. Moreover, previous study reveals that mothers with pre-gestational DM are more likely to have a baby with some congenital malformations compared with normal healthy women who never have diabetes [4]. Some factors are correlated with foetal anomalies in the diabetic mother which include age, obesity, motivation, educational status, duration of the disease, compliance with diet and medications [5]. Furthermore, oxidative markers such as 8-isoprostane and protein carbonyl levels are significantly higher in placentae obtained from women with gestational DM compared to healthy pregnant women. These data demostrate the presence of oxidative stress in the placentae of women with hyperglycaemia which may potentially lead to foetal compromise [6]. Antioxidants prevent or remove oxidative damage to a target molecule through donating an electron to stabilise the free radical [7]. Consumption of natural products with antioxidants such as flavonoid and phenolic acids improves oxidant-antioxidant status and decreases the risk of developing DM complications [8]. Superoxide anion radical (O^−^ _2_) is one of the strongest reactive oxygen species (ROS) among the radicals that are generated after oxygen is taken into living cells. This O^−^ _2_ can change to other harmful ROS, which lead to oxidative stress and implicated in the aetiology of many diseases including DM [9].

Brazilian propolis at doses of 100, 200 and 300 mg/kg body weight for 40 days improve body and kidney weights, serum glucose, lipid profile, malondialdehyde (MDA) and renal function in diabetic male rats [10]. Saudi Arabian propolis at 200 mg/kg/day reduces fasting blood glucose (FBG) and MDA levels, and preserves pancreatic islet of Langerhans in diabetic rats and alleviates diabetic signs [11]. Turkey propolis at doses of 0.2, 0.4 and 0.6 mg/ml per day for 28 days significantly improve cryopreservation and fertilization ability in fish semen [12]. Malaysian propolis at 300 mg/kg/day for 4 weeks has shown antihyperglycaemic property in diabetic rats [13]. However, to date, it is known whether propolis may also ameliorate hyperglycaemia-induced impaired pregnancy outcomes and placental oxidative stress in DM. Hence, the objective of the study was to determine the effects of propolis alone or combined with insulin on maternal status, pregnancy outcomes and placental oxidative stress status in streptozotocin-induced diabetic female rats.

## Methods

### Propolis preparation

Raw propolis of stingless bee (*Heterotrigona itama*) was purchased from the local beekeeper in Kelantan, Malaysia which was collected during dry season (April–June in 2015) and stored at − 40 °C. Briefly, raw propolis (30 g) was washed with distilled water, frozen at − 80 °C and ground into powder. Then, it was mixed vigorously with 100 mL of 70% (*v*/v) ethanol at room temperature on a shaker for 6 h daily for 7 days. It was then filtered, concentrated and freeze-dried or lyophilized to remove ethanol and water to obtain propolis sample to be used in the study [13].

### Experimental design

Forty female Sprague Dawley rats of age 8 to 10 weeks (190–220 g) were used. The animals were obtained from the Laboratory Research Unit, Health Campus, Universiti Sains Malaysia. They were exposed to 12-h light, 12- h dark cycle at 22–24 °C, and given water and food ad libitum. The animals were randomly assigned into 5 groups (*n* = 8 rats per group) as follows: (i) non-DM group (Healthy rats on 1 mL distilled water orally), (ii) DM group (Diabetic rats on 1 mL distilled water orally) (iii) DM + Propolis (Diabetic rats on 300 mg/kg/day propolis orally) (iv) DM + insulin group (Diabetic rats on 5.0 IU/kg/day insulin subcutaneously) and (v) DM + Combined group (Diabetic rats on 300 mg/kg/day propolis orally and 5.0 UI/kg/day subcutaneously). DM was induced using single intraperitoneal injection of streptozotocin (STZ; Sigma-Aldrich Co., St Louis, USA) at 60 mg/kg body weight after fasting for 16 h overnight. Diabetes was confirmed after 48 h of STZ injection [13]. Animals with FBG level of ≥200 mg/dl (Digital Glucometer, Lifescan Inc. Milpitas, USA) were considered as having DM and used. All treatments were given for 4 weeks. After a week of treatment, each female rat was mated overnight at pro-oestrous with a proven fertile male rat to achieve pregnancy and sperm positive smear was considered as Day 1 of pregnancy. Initial and final FBG levels, conception period and maternal weight gain were recorded.

On Day 21 of pregnancy, all female rats were fasted overnight, anaesthetised by intraperitoneal injection of 90 mg/kg ketamine and 5 mg/kg xylazine. Laparotomy was performed on each rat and blood was taken from the heart for biochemical analysis. Then the rats were sacrificed by cervical dislocation. Pregnancy outcomes such as percentages of resorption and implantation loss, number of live and dead foetuses, foetal and placental weights and foetal fasting blood glucose level were assessed. Placental oxidative stress status such as MDA and protein carbonyl (PCO), and total antioxidant capacity (TAC) were assessed using commercial kits (BioAssay Systems. California, USA).

This study was approval by the Animal Ethics Committee of Universiti Sains Malaysia [Animal Ethics Approval/2013/(90)(503) USM] and carried out in accordance with the National Institute of Health Guideline for the care and use of laboratory animals.

### Statistical analysis

All numerical data are expressed as mean and standard deviation (SD). Statistical analysis was performed using Instat. Exe version 3.1. (Charleswork Publishing Services Ltd. Deighton, Huddersfield, UK). One way analysis of variance (ANOVA) followed by Turkey-Kramer test was used to assess the differences among means for normally distributed data and *P* < 0.05 was considered as statistically significant.

## Results

### Effects on maternal parameters

Final FBG level was significantly higher in DM compared with non-DM group. The final FBG levels in DM + Propolis, DM + Insulin and DM + Combined groups were significantly lower compared with DM group but significantly higher than non-DM group. However, the final FBG in DM + Combined group was significantly lower than DM + P group (Table 1).

**Table 1:** Effects of administration of propolis on fasting blood glucose level and conception period of pregnant streptozotocin-induced diabetic rats

Groups	Initial FBG level (mg/dL)	Final FBG level (mg/dL)	Conception period (hours)
Non-DM	89.25 (1.75)	89.88 (0.64)	27.00 (8.49)
DM	404.13 (28.72)^a^	568.00 (48.07)^a^	81.00 (54.33)^a^
DM + Propolis	415.38 (11.87)^a^	274.13 (40.66)^a,b^	42.00 (27.96)
DM + Insulin	413.75 (14.48)^a^	242.13 (61.48)^a,b^	42.00 (27.96)
DM + Combined	424.63 (21.78)^a^	191.88 (18.50)^a,b,c^	33.00 (17.86)^b^

Data are presented as mean (standard deviation), (*n* = 8 per group). DM: diabetes mellitus, Combined: Propolis and insulin, FBG: fasting blood glucose. ^a^*P* < 0.05 compared with negative control, non-DM group; ^b^*P* < 0.05 compared with DM group; ^c^*P* < 0.05 compared with DM + Propolis group (ANOVA followed by Turkey-Kramer post-hoc test)

Conception period was significantly longer in DM group compared with non-DM group. It was significantly shorter in DM + Combined group compared with DM group. There were no significant differences for conception period in DM + Propolis, DM + Insulin and DM + Combined groups compared to non-DM groups (Table 1). Maternal body weight gain was significantly lower in DM and DM + Propolis groups compared with non-DM group. However, the maternal body weight gain was significantly higher in DM + Propolis, DM + Insulin and DM + Combined groups compared with DM group (Table 2).

**Table 2:** Effect of administration of propolis on maternal body weight gain of pregnant streptozotocin-induced diabetic rats

Groups	Maternal body weight gain (g)
Non-DM	155.25 (37.06)
DM	61.00 (16.40)^a^
DM + Propolis	111.75 (33.55)^a,b^
DM + Insulin	112.75 (27.00)^b^
DM + Combined	140.88 (29.67)^b^

Data are represented as mean (standard deviation), (*n* = 8 per group). DM: diabetes mellitus, Combined: Propolis and insulin. ^a^*P* < 0.05 compared with non-DM group, ^b^*P* < 0.05 compared with DM group (ANOVA followed by Turkey-Kramer post-hoc test)

### Effects on pregnancy outcomes

Pre- and post-implantation losses were significantly higher while total number of foetuses was significantly lower in DM group compared with non-DM group. However, pre-implantation loss was significantly lower in DM + Insulin and DM + Combined groups compared to DM group. Post-implantation loss was significantly lower while total number of foetuses was significantly higher in DM + Propolis, DM + Insulin and DM + Combined groups compared to DM group (Table 3). All foetuses exhibited normal physical characteristics with no gross congenital abnormality. No protrusion of any of the organs, and no signs of cleft were seen in lips and palate, and all the foetuses showed normal size, shape and position of tail.

**Table 3:** Effects of administration of propolis on implantation loss and number of foetus of pregnant streptozotocin-induced diabetic rats

Groups	Pre-implantation Loss (%)	Post-implantation Loss (%)	Number of foetus (n)
Non-DM	9.82 (7.80)	8.85 (5.78)	12.50 (1.69)
DM	38.69 (18.93)^a^	42.14 (21.55)^a^	5.25 (2.82)^a^
DM + Propolis	21.44 (12.53)	11.50 (7.82)^b^	9.75 (2.61)^b^
DM + Insulin	19.87 (7.52)^b^	14.45 (16.05)^b^	9.13 (1.81)^b^
DM + Combined	19.88 (10.36)^b^	14.57 (17.38)^b^	9.13 (3.87)^b^

Data are presented as mean (standard deviation), (*n* = 8 per group). DM: diabetes mellitus, Combined: Propolis and insulin. ^a^*P* < 0.05 compared with non-DM group, ^b^*P* < 0.05 compared with DM group (ANOVA followed by Turkey-Kramer post-hoc test)

### Effects on Foetal blood glucose level and body weight

Foetal body weight was significantly lower in DM and DM + Propolis groups compared with non-DM group. It was significantly higher in DM + Combined group compared with DM group. Foetal blood glucose was significantly higher in DM, DM + Propolis and DM + Insulin groups compared with non-DM group. Blood glucose was significantly lower in DM + Propolis, DM + Insulin and DM + Combined groups compared with DM group (Table 4).

**Table 4:** Effects of administration of propolis on foetal body weight and blood glucose level of pregnant streptozotocin-induced diabetic rats

Groups	Foetal body weight (g)	Foetal blood glucose level (mg/dL)
Non-DM	5.37 (0.34)	98.88 (10.95)
DM	4.31 (0.61)^a^	429.13 (118.74)^a^
DM + Propolis	4.66 (0.52)^a^	216.00 (50.24)^a, b^
DM + Insulin	4.83 (0.36)	201.75 (56.94)^a, b^
DM + Combined	5.07 (0.51)^b^	148.63 (33.26)^b^

Data are presented as mean (standard deviation), (*n* = 8 per group). DM: diabetes mellitus, Combined: Propolis and insulin. ^a^*P* < 0.05 compared with non-DM group, ^b^*P* < 0.05 compared with DM group (ANOVA followed by Turkey-Kramer post-hoc test)

### Effects on placental weight and oxidative stress status in diabetic rats

No significant differences in relative placental weight were evident between all the experimental groups (Table 5). Placental MDA and PCO levels were significantly higher while TAC level was significantly lower in DM group compared with non-DM group. However, MDA and PCO levels were significantly lower while TAC level was significantly higher in DM + Propolis, DM + Insulin and DM + Combined groups compared with DM group. TAC was significantly higher in DM + Combined group compared with DM + Insulin group (Table 6).

**Table 5:** Effect of administration of propolis on relative placental weight of pregnant streptozotocin-induced diabetic rats

Groups	Relative placental weight (%)
Non-DM	0.170 (0.023)
DM	0.154 (0.018)
DM + Propolis	0.165 (0.025)
DM + Insulin	0.167 (0.005)
DM + Combined	0.163 (0.018)

Data are presented as mean (standard deviation), (*n* = 8 per group). DM: diabetes mellitus, Combined: Propolis and insulin. No significant difference observed between all groups (ANOVA)

**Table 6:** Effects of administration of propolis on placental oxidative stress markers of pregnant streptozotocin-induced diabetic rats

Groups	Malonaldehyde (nmol/mg protein)	Protein carbonyl (nmol/mg protein)	Total antioxidant capacity (nmol/mg protein)
Non-DM	0.844 (0.173)	1.196 (0.039)	0.393 (0.046)
DM	3.322 (0.442)^a^	3.510 (0.331)^a^	0.158 (0.029)^a^
DM + Propolis	1.147 (0.456)^b^	2.087 (0.339)^a, b^	0.249 (0.028)^a, b^
DM + Insulin	1.116 (0.220)^b^	2.496 (0.428)^a, b^	0.215 (0.018)^a, b^
DM + Combined	1.026 (0.338)^b^	1.967 (0.371)^a,b,c^	0.295 (0.040)^a,b,d^

Data are presented as mean (standard deviation), (n = 8 per group). DM: diabetes mellitus, Combined: Propolis and insulin, FBG: fasting blood glucose. ^a^*P* < 0.05 compared with non-DM group; ^b^*P* < 0.05 compared with DM group; ^c^*P* < 0.05 compared with DM + Propolis group, ^d^*P* < 0.05 compared with DM + Insulin group (ANOVA followed by Turkey-Kramer post-hoc test)

## Discussion

In this study, we investigated the effects of propolis alone as monotherapy or in combination with insulin on maternal status, pregnancy outcomes and placental oxidative stress status in diabetic female rats. Considering that DM is a disorder of multiple etiologies, monotherapy with an antihyperglycaemic agent may not be effective [14]. Initial FBG levels of all the STZ-induced diabetic rats were greater than 200 mg/dL and significantly higher than non-DM group prior to the commencement of treatments showing the establishment of diabetic animal model. Final FBG in DM + Propolis group was comparable with DM + insulin group which may indicate that propolis and insulin are equally effective to produce a comparable antihyperglycaemic effect. The antihyperglycaemic effect of 300 mg/kg/day Malaysian propolis for 4 weeks found in the present study is in line with the findings on propolis from other countries. For instance, Nigerian propolis at doses of 200 and 300 mg/kg/day for 4 weeks [15] and Saudi Arabian propolis at a dose of 300 mg/kg/day for 2 weeks significantly reduce FBG level in DM male rats [11]. Moreover, Egyptian and Brazilian propolis at the dose of 300 mg/kg/day [16] and 900 mg/kg/day [17], respectively, for 2 weeks significantly reduce FBG level in DM male rats, which further support propolis as a potential antihyperglycaemic agent possibly due to the ability of polyphenols in propolis to stimulate insulin secretion by the remaining islets cells with normal function [11]. Furthermore, the antihyperglycaemic effect was found to be more pronounced in DM + Combined group compared with DM + propolis and DM + Insulin groups suggesting that propolis in combination with insulin may produce greater antihyperglycaemic effect than propolis or insulin alone as mono-therapy.

DM may alter reproductive functions in human subject [18]. The longer conception period in DM group compared to non-DM group may be due to the presence of hyperglycaemia which is associated with reduced libido as previously shown in DM male rats [19]. However, the conception period was significantly lower in DM + Combined group, not in DM + Propolis and DM + Insulin groups, compared with DM group. These findings may indicate that propolis combined with insulin is able to improve conception period which could be attributed to the concomitant greater improved FBG level found in DM + Combined group. The significant improved maternal body weight gain in DM + propolis, DM + Insulin and DM + Combined groups could also be attributed to the antihyperglycaemic effect of propolis and insulin. The improved maternal body weight in DM + propolis is in accordance with previous study [13] and propolis has been shown to improve insulin level in diabetic rats [11]. This in turn may improve maternal body weight as insulin is known to exert an anabolic effect through stimulating protein synthesis and inhibiting protein degradation and lipolysis [20].

The significantly higher implantation losses might explain for the lower number of foetus found in DM group compared to non-DM group. These findings support the fact that chronic exposure to hyperglycaemia during early pregnancy is associated with implantation loss [21]. Nevertheless, failure of embryo implantation may cause morphological alterations of embryo that eventually interferes with the implantation and promotes embryo lethality. Additionally, failure of implantation is also resulted from disturbance in tubal transport and subsequent interference in the time of blastocyst arrival in the uterus [22]. These findings support the fact that DM in the first trimester of pregnancy is detrimental to blastocyst and embryo formation [23]. Pre-implantation loss was significantly lower in DM + insulin and DM + Combined group compared to DM group which may suggest that propolis in combination with insulin has greater effect in reducing pre-implantation loss as compared to propolis alone. However, the significantly improved post-implantation loss and number of foetus might suggest the comparable action of propolis, insulin or in combination possibly via their antihyperglycaemic effect to prevent post-implantation loss in this diabetic animal model. These findings are similar with a study using an essential acid taurine that significantly improves pregnancy outcomes and protects both the dam and embryos during pregnancy in diabetic rat [23]. The findings on normal gross morphology of the foetuses are in contrast with a few studies in which prenatal stress in rodents is associated with the risk of developing congenital abnormalities [24]. The external evaluation in the present study might suggest that propolis does not cause any foetal gross malformation and assessment on internal organs is, however, suggested in further study.

Foetal blood glucose level was significantly higher in DM group and this could be associated with STZ-induced maternal hyperglycaemia and hypoinsulinaemia despite normal beta cells function in the foetus. However, foetal blood glucose was significantly improved when treated with propolis or insulin without significant change in foetal body weight. The reduction in foetal birth weight in diabetes may be associated with the detrimental effect of maternal hyperglycaemia leading to intrauterine growth retardation [25, 26]. However, both foetal blood glucose and foetal body weight were significantly improved in DM + Combined group which could be attributed to the improved maternal FBG and weight.

Placental MDA and PCO levels were significantly lower while placental TAC level was significantly higher in DM + propolis, DM + Insulin and DM + Combined groups compared to DM group which may suggest the ability of propolis and insulin to reduce placental oxidative stress. This finding may support previous study showing the antioxidant potential of Iranian propolis in DM rats [27] and the greater beneficial effects in DM + Combined group may suggest the synergistic effect of propolis and insulin. The reduced placental oxidative stress could be indirectly due to the antihyperglycaemic action of propolis and insulin as there is a presence of placental oxidative stress in mother with hyperglycaemia [6]. Apart from that, the antioxidant effect of propolis found in the present study could also partly due to the direct synergistic actions of flavonoids and phenols, which have antioxidant property, that present in Malaysian propolis [28].

## Conclusion

In conclusion, this study may suggest the protective effects of propolis against DM-induced impaired pregnancy outcomes and placental oxidative stress with greater effects when combined with insulin which could be partly due to the synergistic effect of some of its phytochemical constituents. Apart from supporting the traditional belief on the beneficial effect of propolis on health, further studies are suggested to determine its molecular mechanism of action and its possible role in protecting or reducing complications of DM in other organs.

## Abbreviations

ANOVA: One way analysis of variance
DM: Diabetes mellitus
FBG: Fasting blood glucose
MDA: Malondialdehyde
O-2: Superoxide anion radical
PCO: Protein carbonyl
SD: Standard deviation
STZ: Streptozotocin
TAC: Total antioxidant capacity
